# Traditional Dance Improves the Physical Fitness and Well-Being of the Elderly

**DOI:** 10.3389/fnagi.2019.00075

**Published:** 2019-04-05

**Authors:** Styliani Douka, Vasiliki I. Zilidou, Olympia Lilou, Vasiliki Manou

**Affiliations:** ^1^School of PE and Sport Science, Department of Physical Activity and Recreation, Aristotle University of Thessaloniki, Thessaloniki, Greece; ^2^Laboratory of Medical Physics, Medical School, Aristotle University of Thessaloniki, Thessaloniki, Greece; ^3^School of PE and Sport Science, Department of Human Performance, Aristotle University of Thessaloniki, Thessaloniki, Greece

**Keywords:** greek traditional dance, elderly, physical health, physical function, well-being

## Abstract

Regular physical activity is considered one of the most important factors for lifestyle, for maintaining good health in older ages and increasing life expectancy. Dance is considered an activity that involves coordinating movements with music, as well as brain activation because it is constantly necessary to learn and remember new steps. Dance as a musical-kinetics skill, requires the coordination of body movements with rhythmic stimuli, developing the adaptability of the movement. One-hundred-thirty (130) elderly people aged 60 years and over (mean age 67 years old) with an average of 8 years of education, attended Greek traditional dance sessions for 32 weeks. The frequency was 2 times per week, for 75 min per session. Dances were selected from all over Greece with moderate intensity initially. During the program, they had the opportunity to try with greater intensity dances. At the beginning and after the end of intervention all the participants were evaluated by the Fullerton Senior Fitness Test for their physical fitness, the Single Leg Balance and the Handgrip Strength Test. The results showed a significant improvement in their physical fitness (Chair Stand: *T* = −5.459, *p* < 0.001; Arm Curl: *T* = −5.750, *p* < 0.001; Back Scratch: *T* = −4.648, *p* < 0.001; Sit and Reach: *T* = −4.759, *p* < 0.001; 2 min Step: *T* = −5.567, *p* < 0.001; Foot Up and Go: *T* = −8.599, *p* < 0.001) and at their static balance with eyes open (Balance 1 leg: *T* = −4.996, *p* < 0.001) and Handgrip Strength (Handgrip: *T* = −3.490, *p* < 0.001). Elderly seem to enjoy dancing as an activity while maintaining their functionality. Probably the elderly in traditional dance cause prosperity in their lives by promoting active aging.

## Introduction

The percentage of people aged 60 and over is growing faster worldwide than any other age group, and the resulting aging population presents challenges and opportunities for all countries increased due to new social and economic demands. Countries adapting to this changing demographic, invest in healthy aging to enable people to live longer and have a healthy life. Healthy Ageing involves creating an environment that allows people to engage actively throughout their lives. Both the elderly and the environments in which they live are diverse, dynamic, changing and playing an important role in determining the physical and mental ability throughout a person’s life. In interaction with each other, they possess incredible possibilities to allow or limit healthy aging (World Health Organisation, 2018).

The advanced age besides changes in physical fitness, increases sensitivity to chronic diseases and disabilities, and reduces the quality of life (Wanderley et al., 2015). In many studies it has been shown that the combination of exercise with nutrition is considered effective intervention for elderly people. Improving or maintaining their nutritional status combined with exercise is associated with many benefits, including increased physical fitness and strength, reducing the incidence of sarcopenia, reducing functional loss and rehabilitation of musculoskeletal injuries, reducing the risk of falls and/or their frequency. Also, improving gait and balance, their quality of life and mortality and morbidity of diseases by 30% of all causes (Weening-Dijksterhuis et al., 2011; Cadore et al., 2013). The satisfaction of life is observed as a basic characteristic of well-being (Fugl-Meyer et al., 2002), constitutes a provision for physical health (Dominick et al., 2002) and has gradually entered a more central in healthcare systems (Fugl-Meyer et al., 2002; Daig et al., 2009).

Regular physical activity is considered one of the most important factors for lifestyle, maintaining good health in older ages and increasing life expectancy (Lee et al., 2012). In surveys with elderly people, it seems that physical exercise may have beneficial effects on cognitive (Lautenschlager et al., 2008) and the physical functions (Villareal et al., 2011). In addition, physical activity in the elderly is associated with increased survival (Manini et al., 2006; Stessman et al., 2009). In another study it seems that physical activity when done regularly, delaying the reduction of functional abilities associated with aging and sometimes reverses the loss and morbidity (Nied and Fraklin, 2002). The activities proposed for the elderly should lead to the improvement or maintenance of physical and mental health (Stathi et al., 2004).

Alternative categories of exercise programs have been performed in elderly people (Hui et al., 2009; Sofianidis et al., 2009). Dance of any type is being used for many years as a treatment modality. Dance involves elderly people and increases their motivation (Lima and Vieira, 2007). Furthermore, it has been shown in investigations that elderly people are excited when participating in dance programs, thus improving the quality of life, balance and mobility (Song et al., 2004; Federici et al., 2005).

Dance is considered an activity that offers the involvement of different senses and connects movement to music with self-expression and applies different aspects of personality (Kaufmann, 2011; Studer-Lüthi and Züger, 2012). Music, which is an important component of dance, improves physical performance. It’s easier going to start moving, walking, dancing or to deal with any kind of exercise if some people choose their favorite music. Reduces fatigue and increases the levels of psychological stimulation during exercise (Jing and Xudong, 2008). Kattenstroth et al. (2011) showed that elderly people dancing on a regular basis have better balance, postural stability, flexibility and physical reaction time. Alpert et al. (2009) showed progressive balance enhancement in the sensory organization test (SOT) in their study for 13 women aged 52–88 years performing jazz dance that lasted 15 weeks. Also, Hui et al. (2009) shown that after 24 sessions for 52 adults aged 68 on average and trained in low-impact aerobic dancing, the dancers had improved their dynamic balance in Time Up-and-Go test, but not their static balance. Moreover, dance has been proposed as an actual promising program for the development and improvement of balance and to prevent falls in the elderly people (American Geriatrics Society, British Geriatrics Society, and American Academy of Orthopaedic Surgeons Panel on Falls Prevention, 2001; Judge, 2003).

Aweto et al. (2012) investigated the effects of dance programs on patients with hypertension with specific cardiac vascular conditions. Hackney and Bennett (2014), attempted to evaluate Parkinson’s disease impact on dance participation as these patients had mainly mobility problems, with an increased risk of falls, reducing their quality of life as a result. Tsimaras et al. (2012) investigated the impact of a Greek dance program in 13 adults with hearing problems in their aerobic capacity and muscle tone. After 12 weeks of dance program observed significant progress in physiological peak parameters such as oxygen consumption and exhaustion time. Other research with Greek traditional dance, performed on people with breast cancer, showed improvement in their physical functioning, satisfaction with their lives as well as reducing depression symptoms (Kaltsatou et al., 2011).

The purpose of this study was to investigate the impact of a Greek traditional dances program on elderly people over the age of 60. Particularly, to investigate whether Greek traditional dance as a form of aerobic exercise, could improve the functional capacity and the well-being of the elderly people.

## Materials and Methods

### Participants

One-hundred-thirty (130) Greek elderly people aged 60 years and over (mean age 67 years old) with an average span of education 8 years (Q2 = 8, IQR = 6), attended Greek traditional dance sessions for 32 weeks. The frequency of the intervention was twice a week, and each session lasted 75 min. The intervention was performed at the Greek Association of Alzheimer Disease and Relative Disorders (Alzheimer Hellas) and at the Day Care Centers of Municipality of Thessaloniki, Greece. During the program, they had the opportunity to try with greater intensity dances. In the period of 32 weeks, the maximum number of sessions to complete the intervention was 64 and the average presence of the participants was 51 sessions for completing the intervention.

Moreover, we recruited a control group (20 individuals) that it is matched to a smaller group of intervention participants (20 individuals) in demographic and baseline somatometric data. The control group did not receive any type of training and thus it was a waiting group.

For their participation in the research, they had to be in a good functional and emotional condition and to not participate in another dance program. Each of them was required to be examined by a doctor to ensure their participation in a mild intensity activity. In addition, elderly who have been diagnosed with hypertension, cardiac and respiratory failure were excluded from the intervention program. Also, the elderly who did not complete at least 80% of the total attendance hours of the program was rejected by this.

Written consent was requested for their participation in this study, after being given the required explanations for its purpose. At the beginning and after the end of intervention all the participants were evaluated for their fitness and functional capacity by a fitness instructor. Ethical and Scientific Committee of GAARD approved the protocol of this study. Also, the participants signed their acceptance of viewing videos and posting photos related to the traditional dance intervention program in online and/or print media for scientific purposes and public information.

### Outcome Measures

#### Physical Assessments

All participants were evaluated both at the beginning of the intervention and after the end of this to investigate any improvement caused by the intervention. They were evaluated on their physical fitness and functional capacity by the Senior Fitness Fullerton Test. This test is safe, it does not require to use any special equipment and is used to assess six parameters such as strength, flexibility, coordination and endurance. Specifically, the test battery consists of: 30-s chair stand, arm curl, chair sit-and-reach, back scratch, 2-min step-in-place, and 8-foot (2.44-m) up and go (Rikli and Jones, 1999). Furthermore, they were evaluated on their static balance by the Stork Balance test (Johnson and Nelson, 1969).

Handgrip strength was measured using a hydraulic hand dynamometer holding the dynamometer in the dominant hand (Saehan Corp., Masan, Korea). Three trials from the dominant hand were calculated and used the best for the analysis. Handgrip strength is expressed in kilograms (kg). Furthermore, the jumping vertical ability was evaluated by free hand countermovement jump using OptoJump system (Microgate, Bolzano, Italy) and the jumping was calculated in centimeters. Lastly, the body mass index (BMI) was calculated following the measurement weight and height of each participant.

#### Greek Traditional Dances

**Figure 1:**
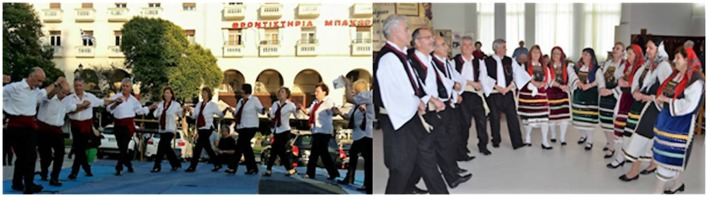
Participants in a Greek traditional dance named: Litos and Zonaradikos. They are dancing in the regions of Macedonia and Thrace of Greece.

Dances were selected for the intervention in the present study were from all over Greece (Figure 1) and ranked in three categories: mild, moderate and high intensity. Further classification of the dances was carried out in relation to the complexity and the number of steps, the position of hands, as well as the intensity of the rhythm. After the integration of 2–3 sessions, most of the dances were selected were considered, changing progressively and increasing, indicative of the age and physical abilities of the elderly. Initially, the dances had few and simple steps, for example, 6-step dances with minimal hand movement. The increasing difficulty, resulting in the higher intensity, was applied with dances of more steps, 8–16 steps, combined with alternations in the movements of the hands and body of the participants.

### Statistical Analysis

#### Demographics

Demographic data (age, education level, etc.) was first tested for normality assumption using visual inspection of histograms, normal Q-Q plots and boxplots, in terms of skewness and kurtosis as well as using the normality tests (Shapiro-Wilk test) in order to calculate proper descriptive statistics. Since the age and the education level of participants were not approximately normally distributed median and interquartile range were calculated. The proportion of male/female participants was also computed. Similar procedures were followed when analyzing demographic data in a subgroup of intervention participants and matched controls.

#### Data

Participants underwent an evaluation of physical fitness both before and after the intervention. The assumptions of repeated measures analysis of variance (ANOVA) were not fulfilled and as such, score differences were computed in two-time conditions (post-pre) then explored for normality assumption. Subsequently, we performed Wilcoxon signed-rank tests as the score differences of the tested parameters were not approximately normally distributed. Statistical analysis was performed using the IBM SPSS Statistics (Version 23) and defining setting the significance level (a) to 0.05. Bonferroni correction was used to counteract the problem of alpha inflation due to multiple comparisons.

In the second analysis, we aimed to explore the within changes in each group as well as the between-group differences. Initially, we compared the performance of two groups in the baseline physical assessment performing Mann-Whitney *U* test or independent samples *t*-test depending on the normality assumption. Subsequently, the assumptions of Mixed Model ANOVA were not fulfilled and as such, an alternative analysis design was followed. More precisely, within-group changes were investigated using Wilcoxon signed-rank tests after grouping data by the group while between-groups score differences were compared using Mann-Whitney *U* test. Moreover, we performed Bonferroni correction to counteract the problem of multiple comparisons.

## Results

### Demographics

Demographic data of 130 elderly participants are described in the following table (Table 1).

**Table 1:** Demographic data of elderly participants as age, education years and gender.

Age Median, [Q1, Q3]	Education Median, [Q1, Q3]	Gender Male/Female
67.00, [63.00, 71.00]	8.00, [6.00, 12.00]	23/107 17.69%/82.31%

The subgroup of the intervention group did not significantly differ in age, education and BMI relatively to the matched controls [Age—Dance subgroup: 66.50, (62.00, 73.00), Controls: 65.50, (61.00, 69.50), *U* = 181.50, *p* = 0.615; Education—Dance subgroup: 6.00, (6.00, 11.25), Controls: 6.50, (6.00, 11.25), *U* = 184.00, *p* = 0.643; BMI—Dance subgroup: 29.10 (3.64), Controls: 27.76 (3.06), *t*_(38)_ = 1.263, *p* = 0.214].

### Physical Assessments

Planned comparisons of physical assessments test scores in two-time points (before and after the training) showed that neither the height (*W* = −0.258, *p* = 0.796) and the weight (*W* = −0.074, *p* = 0.941) nor the BMI parameter (*W* = −0.186, *p* = 0.852) changed.

**Figure 2:**
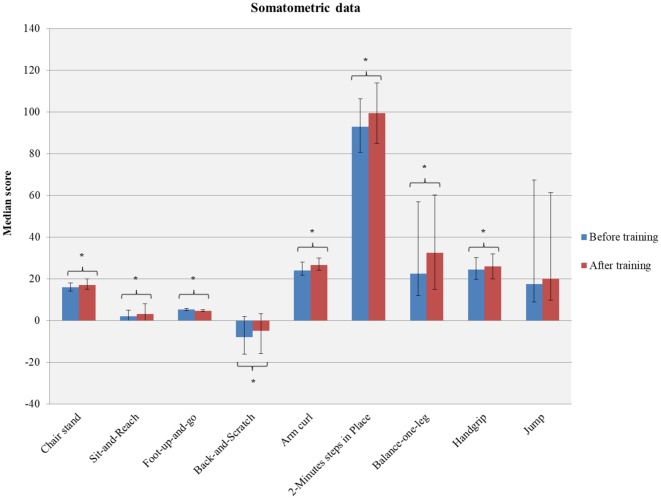
A summary of intervention-induced changes in different physical fitness components. *indicates the significant improvement in the participants of these tests after the dance intervention.

However, the participants’ performance significantly altered in most of the physical tests tasks (Chair Stand: *W* = −5.459; *p* < 0.001; Sit and Reach: *W* = −4.759, *p* < 0.001; Foot Up and Go: *W* = −8.599, *p* < 0.001; Back Scratch: *W* = −4.648, *p* < 0.001; Arm Curl: *W* = −5.750, *p* < 0.001; 2 min Step: *W* = −5.567, *p* < 0.001; Balance 1 leg: *W* = −4.996, *p* < 0.001; Handgrip: *W* = −3.490, *p* < 0.001) apart from the jump ability (*W* = −0.954, *p* = 0.340; Table 2; Figure 2).

**Table 2:** The intervention provoked improvement in almost all the physical fitness parameters.

Physical fitness	Before training Median, [Q1, Q3]	After training Median, [Q1, Q3]	Score change Median, [Q1, Q3]	Test results
Chair Stand	16.00, [14.00, 18.00]	17.00, [15.00, 20.00]	1.00, [0.00, 3.00]	*W* = −5.459, *p* < 0.001
Sit and Reach	2.00, [0.00, 5.00]	3.00, [0.00, 8.00]	2.00, [0.00, 5.00]	*W* = −4.759, *p* < 0.001
Foot Up and Go	5.29, [4.64, 5.85]	4.71, [4.27, 5.17]	−0.45, [−0.93, −0.19]	*W* = −8.599, *p* < 0.001
Back Scratch	−8.00, [−16.25, 2.00]	−5.00, [−16.00, 3.25]	2.00, [−1.00, 4.00]	*W* = −4.648, *p* < 0.001
Arm Curl	24.00, [21.75, 28.00]	26.50, [24.00, 30.00]	2.00, [0.00, 4.00]	*W* = −5.750, *p* < 0.001
2-min step	93.00, [80.75, 106.25]	99.50, [85.00, 114.00]	6.00, [2.00, 13.25]	*W* = −5.567, *p* < 0.001
Balance-1-leg	22.40, [12.02, 56.87]	32.44, [14.99, 60.28]	3.79, [0.29, 15.20]	*W* = −4.996, *p* < 0.001
Handgrip	24.50, [19.75, 30.25]	26.00, [20.00, 32.00]	1.00, [−1.00, 4.00]	*W* = −3.490, *p* < 0.001
Jump ability	17.48, [8.87, 67.50]	20.00, [9.70, 61.25]	1.00, [−3.23, 3.00]	*W* = −0.954, *p* = 0.340

The intervention seems to promote significant improvement in Chair stand task as test scores after the training were increased compared to the baseline [pre-training: 16.00, (14.00, 18.00); post-training: 17.00, (15.00, 20.00)]. In more detail, 82 out of 130 participants increased their Chair Stand score, 28 participants did not show any significant change while 20 out of 130 decreased their performance at the aforestated task. Thus, a Wilcoxon signed-rank test determined that there was a statistically significant median increase of 1.00, [0.00, 3.00] in scores at Chair Stand task.

Similar findings were revealed in Sit and Reach task. A significant increase of 2.00, [0.00, 5.00] was observed when comparing test scores at the two-time points [pre-training: 2.00, (0.00, 5.00); post-training: 3.00, (0.00, 8.00)]. More particularly, 83 out of 130 showed enhanced scores at the post-training relative to the baseline, 30 participants decreased their scores while the scores of 17 participants preserved at the two-time points.

Moreover, a Wilcoxon signed-rank test determined that there was a statistically significant median decrease of 0.45, [−0.93, −0.19] in scores at Foot-Up-and-Go task [pre-training: 5.29, (4.64, 5.85); post-training: 4.71, (4.27, 5.17)]. In the Foot-Up-and-Go task 121, out of 130 participants decreased their scores and nine participants showed increased score after the training compared to the baseline. Significant improvement was observed at the Back and Scratch test as a median increase of 2.00, [−1.00, 4.00] [pre-training: −8.00, (−16.25, 2.00); post-training: −5.00, (−16.00, 3.25)]. Eighty-two participants enhanced their scores after training, 15 participants preserved their performance while 33 out of 130 participants showed a decreased in their scores at the Back and Scratch task.

A significant median increase of 2.00, [0.00, 4.00] was observed in Arm curl task when comparing scores both before and after the intervention [pre-training: 24.00, (21.75, 28.00); post-training: 26.50, (24.00, 30.00)]. Ninety-one participants increased their Arm Curl scores, 24 participants showed the opposite finding whereas 15 participants did not change their performance. Moreover, the intervention seems to promote gains in 2-min steps in the place as a median enhance of 6.00, [2.00, 13.25] [pre-training: 93.00, (80.75, 106.25); post-training: 99.50, (85.00, 114.00)]. More precisely, 104 out of 130 participants improved their performance at the 2-min steps in the place task, 25 participants decreased their scores and only one did not show any change.

Ninety-nine out of 130 participants improved their balance as indicated by their enhanced scores at the Balance-1-leg task after the intervention while 31 decreased their performance at the same task when comparing the scores at the two time-points. Furthermore, we observed a significant increase of 3.79, [0.29, 15.20] after the intervention compared to the baseline (pre-training: 22.40, [12.02, 56.87]; post-training: 32.44, [14.99, 60.28]). Additionally, the intervention given seems to induce positive gains in Handgrip task. Sixty-nine participants enhanced their performance after the training compared to the baseline evaluation, 34 had the opposite finding while 27 out of 130 participants remained stable. A significant increase of 1.00, [−1.00, 4.00] was found in Handgrip scores comparing participants’ performance both before and after the training [pre-training: 24.50, (19.75, 30.25); post-training: 26.00, (20.00, 32.00)].

Although subjects showed an increase in their scores at the Jump ability [pre-training: 17.48, (8.87, 67.50); post-training: 20.00, (9.70, 61.25)], score change did not reach statistical significance.

A summary of the aforementioned results is displayed in Table 2 and Figure 2.

**Figure 3:**
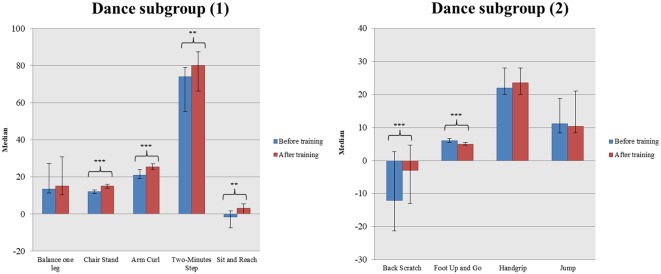
Alterations in different physical assessment parameters in a dance subgroup (*** denotes *p* ≤ 0.001, ** denotes *p* ≤ 0.010).

**Figure 4:**
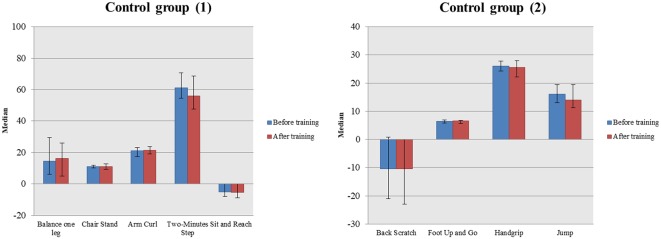
Comparison in somatometric data of the control group in two-time conditions (before and after the waiting period).

In the second analysis we found that the two groups did not have significant differences in their baseline physical performance [all *p*-values (Bonferroni corrected) > 0.05]. Within-group changes were found only in the subgroup of dance group but not in the controls [in all tasks *p*-values (Bonferroni corrected) > 0.05]. In more detail, the intervention group showed significant improvement, when comparing their scores before and after training, in most tasks of physical assessment such as the Chair Stand [pre-training: 12.00, (11.00, 13.00); post-training: 15.00, (14.00, 16.00); *W* = −3.737; *p* < 0.001], Arm Curl [pre-training: 21.00, (19.00, 24.00); post-training: 25.50, (24.00, 27.00); *W* = −3.881; *p* < 0.001], 2-min Step [pre-training: 74.00, (55.25, 79.00); post-training: 80.00, (66.25, 87.50); *W* = −3.119; *p* = 0.002], Sit and Reach [pre-training: −1.50, (−7.50, 1.75); post-training: 3.00, (0.00, 5.50); *W* = −3.163; *p* = 0.002], Back Scratch [pre-training: −12.00, (−21.25, 2.75); post-training: −3.00, (−13.00, 4.75); *W* = −3.431; *p* = 0.001] and Foot Up and Go [pre-training: 6.03, (5.53, 6.73); post-training: 4.91, (4.70, 5.54); *W* = −3.509; *p* < 0.001; Figures 3, 4].

Between-group analysis revealed significant differences in the aforementioned tasks in favor of the dance subgroup. More precisely, the intervention subgroup showed greater changes in task scores compared to the control group in the following tasks: Chair Stand [Dance subgroup: 3.00, (2.00, 5.00); Controls: 0.00, (−1.00, 0.00); *U* = 23.50; *p* < 0.001], Arm Curl [Dance subgroup: 4.00, (2.00, 6.00); Controls: 1.00, (−1.00, 1.00); *U* = 28.00; *p* < 0.001], 2-min Step [Dance subgroup: 8.00, (2.50, 15.25); Controls: −3.50, (−9.75, 0.50); *U* = 60.00; *p* < 0.001], Sit and Reach [Dance subgroup: 4.50, (1.00, 8.75); Controls: 0.00, (−.75, 1.00); *U* = 80.00; *p* = 0.001], Back Scratch [Dance subgroup: 3.00, (1.25, 10.75); Controls: −1.00, (−2.00, 0.00); *U* = 46.50; *p* < 0.001] and Foot Up and Go [Dance subgroup: −0.88, (−1.34, −0.57); Controls: −0.08, (−0.38, 0.21); *U* = 47.00; *p* < 0.001].

## Discussion

In the present study, one-hundred-thirty (130) elderly people participated in an intervention program of Greek traditional dances to investigate how their physical fitness and functional capacity were affected, to enable them to have an independent and autonomous life improving their lifestyle. To assess the beneficial role of dance, we investigated the effect of an elderly dance team lasting 32 weeks with a frequency of two times a week for 75 min each.

The results of the post-intervention evaluations of the elderly compared to the initial pre-intervention evaluation showed significant statistical findings. A significant improvement was observed in the strength of the legs (Chair Stand test), a test that assesses the ease of climbing the stairs, walking speed and reduce the risk of falls. This result is consistent with the results in the Bohannon’s (1995) study, where it is mentioned that the ability to stand up from the chair is important because of the correlation that exists with other performance variables such as balance and falls. Traditional dances with the different movements that perform the legs, enhance the strength of the lower limbs resulting in a better balance in the elderly.

Also, a significant improvement was found in the Sit and Reach test which is measure lower back and hamstring flexibility and is important as because tightness in this area is implicated in lumbar lordosis, lower back pain and forward pelvic tilt as well as in the Back-Scratch test which assesses the general shoulder range of motion by measuring how close the hands can be brought together behind the back. In the Sit and Reach test have improved their flexibility by 2.48 cm and in Back-Scratch test have improved their flexibility by 2.07 cm. These significant results of our research are supported by the results of the research by Carvalho et al. (2009), where elderly participated in a program lasting 8 months and showed an improvement of 17.4% in the Sit and Reach test and 14.5% in the Back-Scratch test. It should be noted that an important measure for the prevention of abnormal abilities is considered to be the strength of the lower limbs, as well as the upper body for performing daily activities. The different positions of the hands and the combination of the movement of hands and the rest of the body that require the traditional dances, improved flexibility of participants.

Their aerobic endurance after dance intervention seems to have improved significantly. Some research shows that improvements in the 2-min test may correspond to improvements in cognitive function (Tanne et al., 2005; Stanek et al., 2011). At the Foot Up and Go test, which evaluated dynamic balance and agility, we found statistically significant results. Purath et al.’s (2009) study, showed that after an exercise program with aerobic activity, flexibility, balance and muscle strength, the time of this test decreased, resulting in improved dynamic balance, reaction time and the strength of the lower limbs. According to Duncan and Earhart’s (2014) research, after a period of 24-months of dance intervention, participants had significantly reduced their time in this test, indicating that dance participants had improved over time. This is also observed in the Hamburg and Clair’s (2004) research, where 36 adults aged 63–86 years increased their balance and standing toe/heel lifts as well as their speed and rhythm gait.

A statistically significant improvement was observed in strength by measuring hand grip (in a rate of about 5.5%), presumably in different gestures ranging from dance to dance, as well as in the same dance. The studies have reported the correlation of hand grip with the reduction of health in the elderly, mainly by linking it to functional disability (Onder et al., 2005) and mortality (Rantanen et al., 2003; Al Snih et al., 2004). A small number of studies indicate the correlation between muscle strength and cognition (Alfaro-Acha et al., 2006; Buchman et al., 2007). Participants in traditional dance have improved their time in the test that evaluate the static balance by about 20%. In a related study by Melzer et al. (2005), showed that the group who participated in the three-month balance training program improved its time by 64% compared to another group that participated in a strength program. According to Duncan and Earhart (2014), measurements of static balance showed a steady improvement in the elderly following dance interventions. Also, Sofianidis et al. (2009) reported that the Greek traditional dance seems to be effective in enhancing the static and dynamic balance in 10-weeks intervention.

Keogh et al. (2009) reported that dancing is a type of physical activity that indicates that this particular activity might improve older adults’ lower body bone-mineral content and muscle strength, as well as reduce the prevalence of falls and cardiovascular health risks. Participating in dance may allow the elderly to improve their physical function, health and well-being. Another beneficial advantage is that they can significantly improve their aerobic capacity, lower body muscle endurance, strength and flexibility, balance, agility and gait through the dancing.

Physical activities for the elderly people include both regular and recreational actions in their daily social life (climbing stairs or walking) performing various tasks (for people who continue working), participating in different sports games as well as in specially designed exercise programs, such as the traditional dance. Dance beyond many benefits helps individuals to improve their body posture. Proper posture improves overall balance and generally there are positive effects on the body. Bones are well aligned, the vital organs are properly positioned, and the muscles, joints and ligaments can function in the way they should be. Also, good posture contributes to encouraging the normal functioning of the nervous system and is important for health and general well-being. Lima and Vieira (2007) at their research, showed that after a dance intervention, the elderly had improved skills such as flexibility, balance and coordination but also improved attitude and control of their movements.

In a previous research on people over 60 years, showed that social dance supported efforts to relieve physical and psychological degradation, provided a strong sense of pleasure and continuity as well as a vehicle for the changes required by aging. It also provided a strong sense of community as it allowed participants to showcase their “cultural heritage” (Cooper and Thomas, 2002). Another research with 24 older social dancers (average age 80), showed that after the intervention they had better balance, walked faster and had a longer mean step with a more stable pattern during walking with reduced stance time, longer swing time and shorter double support time (Verghese, 2006).

Furthermore, dance, in addition to physical activity, combines the emotion, social interaction, motor coordination and music, thus creating a thriving environmental condition for individuals. Through the revival of music and dance, the elderly have the opportunity to relive the past through the present. Individuals initiate to understand the meaning of these two, either individually or as a combination, and are benefiting from the positive effects on the body and mind. The importance of social interaction through music places them in a process, so the elderly can share their passion with others. This interaction with other people eliminates the feeling of loneliness and enhances their psychological status. Moreover, it is considered an important fact that their self-esteem and mood increase as they realized they can engage in new skills.

This study demonstrates the effects of traditional dance on people who choose to systematically participate in organized Greek traditional dance lessons as a means of exercise. In the analysis of the control group with a small intervention group, it was observed that in most tests and particularly in Fullerton’s domains, there was an improvement in their performance after the intervention, such as the same was found for all of the 130 participants. However, we should consider that one limitation of the study is the lack of randomization that strengthens the generalization of our results as well as the small number of control subjects recruited.

Greek traditional dance is a physical activity that contributes positively to many factors on the physical health of elderly people by enhancing the well-being outcomes for elderly people. Dance, specifically for the elderly, is a very interesting type of physical activity, because it carries less risk of injury than many other types of exercise. The Greek traditional dance for Greek elderly people is particularly important, because it relates to the tradition, culture, but also their lives. Thus, it is a physical activity more popular and hence readily selectable by the elderly, which can equally have beneficial effects of exercise.

Generally, dance offers a host of physical and mental benefits to individuals, especially when exercised to protect or improve their health. As well, exercise and participation in physical activities are associated with better performance in cognitive functions. Especially, in our previous study, the intervention involved dance training with adaptive difficulty and intensity. The results demonstrated the functional reorganization of cortical rest networks (Zilidou et al., 2018).

## Conclusion

Through this research conducted on Greek traditional dances programs, it is believed that dancing could be defined as an important and effective tool for the prevention and the fight against the health problems of the elderly. The results of this study show that dancing contributes to the well-being of the elderly with a view of independent and quality living. Maintaining their physical fitness and functional capacity at satisfactory levels, lead them to a more qualitative and independent lifestyle while the risk of various diseases is reduced.

## Future Directions

Dance is demonstrating greatly that it improves the elderly’s functional ability and well-being. Therefore, it is suggested in a new study to investigate the physical effects of dance by combining nutritional education and psychological effects and comparing them with individuals of vulnerable groups such as patients with Parkinson disease. In Parkinson’s disease, a healthy diet and exercise are considered important factors to stay healthy and active while maintaining the satisfactory levels their energy.

## Ethics Statement

Ethical and Scientific Committee of GAARD approved the protocol of this study.

## Author Contributions

ZV: designed and implemented the dance program, collected the data, guided the analysis, prepared the initial draft of the manuscript, discussed the results and revised the manuscript. LO: implemented the dance program data and revised the manuscript. MV: contributed the physical assessments. DS: co-guided the study.

## Conflict of Interest Statement

The authors declare that the research was conducted in the absence of any commercial or financial relationships that could be construed as a potential conflict of interest.
